# 1999–2009 Trends in Prevalence, Unawareness, Treatment and Control of Hypertension in Geneva, Switzerland

**DOI:** 10.1371/journal.pone.0039877

**Published:** 2012-06-27

**Authors:** Idris Guessous, Murielle Bochud, Jean-Marc Theler, Jean-Michel Gaspoz, Antoinette Pechère-Bertschi

**Affiliations:** 1 Unit of Population Epidemiology, Division of Primary Care Medicine, Department of Community Medicine, Primary Care and Emergency Medicine, Geneva University Hospitals, Geneva, Switzerland; 2 Community Prevention Unit, University Institute of Social and Preventive Medicine, Lausanne University Hospital, Lausanne, Switzerland; 3 Unit of Hypertension, Departments of Specialties of Medicine and Community Medicine and Primary Care and Emergency Medicine, Geneva University Hospitals, Geneva, Switzerland; Fundación para la Prevención y el Control de las Enfermedades Crónicas No Transmisibles en América Latina (FunPRECAL), Argentina

## Abstract

**Background:**

There are no time trends in prevalence, unawareness, treatment, and control of hypertension in Switzerland. The objective of this study was to analyze these trends and to determine the associated factors.

**Methods/Findings:**

Population-based study conducted in the Canton of Geneva, Switzerland, between 1999 and 2009. Blood pressure was measured thrice using a standard protocol. Hypertension was defined as mean systolic or diastolic blood pressure ≥140/90 mmHg or self-reported hypertension or anti-hypertensive medication. Unawareness, untreated and uncontrolled hypertension was determined by questionnaires/blood pressure measurements. Yearly age-standardized prevalences and adjusted associations for the 1999–2003 and 2004–2009 survey periods were reported. The 10-year survey included 9,215 participants aged 35 to 74 years. Hypertension remained stable (34.4%). Hypertension unawareness decreased from 35.9% to 17.7% (P<0.001). The decrease in hypertension unawareness was not paralleled by a concomitant absolute increase in hypertension treatment, which remained low (38.2%). A larger proportion of all hypertensive participants were aware but not treated in 2004–2009 (43.7%) compared to 1999–2003 (33.1%). Uncontrolled hypertension improved from 62.2% to 40.6% between 1999 and 2009 (P = 0.02). In 1999–2003 period, factors associated with hypertension unawareness were current smoking (OR = 1.27, 95%CI, 1.02–1.59), male gender (OR = 1.56, 1.27–1.92), hypercholesterolemia (OR = 1.31, 1.20–1.44), and older age (OR 65–74yrs vs 35–49yrs  = 1.56, 1.21–2.02). In 1999–2003 and 2004–2009, obesity and diabetes were negatively associated with hypertension unawareness, high education was associated with untreated hypertension (OR = 1.45, 1.12–1.88 and 1.42, 1.02–1.99, respectively), and male gender with uncontrolled hypertension (OR = 1.49, 1.03–2.17 and 1.65, 1.08–2.50, respectively). Sedentarity was associated with higher risk of hypertension and uncontrolled hypertension in 1999–2003.

**Conclusions:**

Hypertension prevalence remained stable since 1999 in the canton of Geneva. Although hypertension unawareness substantially decreased, more than half of hypertensive subjects still remained untreated or uncontrolled in 2004–2009. This study identified determinants that should guide interventions aimed at improving hypertension treatment and control.

## Introduction

Hypertension is one of the major causes of disease burden worldwide [1]. It affects approximately 37–55% of the adult population in Europe [2]. Hypertension is the most important modifiable cardiovascular risk factor for stroke, coronary artery disease, heart failure and end-stage renal disease, and it also increases all-cause mortality [3].

Trends in hypertension prevalence differ according to geographic and population characteristics and definition of arterial hypertension. In the United States, the last NHANES 1988–2008 analysis reported an increase in prevalence from 23.9% in 1988–1994 to 28.5% in 1999–2000. The prevalence remained stable since then [4]. The population-based prevalence of hypertension was also stable (19.7%–21.6%) in Canada between 1992 and 2009 [5]. Compared to North America, European countries have a higher prevalence of hypertension [6]. In Switzerland, the prevalence of hypertension, based on measured blood pressure (BP), varied with age and gender between 20% and 50% [7], [8]. There are no reliable recent trend summaries of hypertension changes (taking into account anti-hypertensive drugs information) from Europe [9].

To control the public health burden of hypertension, several guidelines recommend the screening, treatment and control of high BP [10], [11]. Hypertension awareness, treatment and control have generally increased in the last decades [4], [5], [12], [13]. Yet, the rates of uncontrolled hypertension remain greater than 50% in recent reports [4], [12], [13]. Factors such as smoking, obesity, education, alcohol consumption and age have been associated with the risk of untreated and uncontrolled hypertension [7], [14]–[16].

In Switzerland, there are no recent time trends in hypertension treatment, control and unawareness. We analyzed the 1999–2009 trends from a large ongoing population-based study conducted in Switzerland.

Specifically, the aims of this study were to assess 10-year trends changes in hypertension prevalence, unawareness, treatment and control for the adult population living in the canton of Geneva, Switzerland. We also aimed to identify factors associated with these four outcomes.

## Methods

### Participants

The *‘Bus Santé’* is an ongoing cross-sectional population-based study, which collects information on cardiovascular risk factors, diet and physical activity. Yearly, a representative stratified sample of 500 men and 500 women from the population of the Geneva Canton is recruited and studied [17]. Three stations receive participants. The first two stations are fixed and are based within the Geneva University Hospitals. The third station is a medical mobile unit, which visits three parts of the canton of Geneva. Four trained collaborators interview and examine participants. All procedures are reviewed and standardized across technicians on a regular basis.

Subjects are selected independently throughout each year to represent the canton’s approximately 100’000 male and 100’000 female non-institutionalized residents aged 35 to 74 years. Eligible subjects are identified using a standardized procedure using an annual residential list established by the local government. This listing includes all potential eligible participants except persons living illegally in the country. Stratified random sampling based on the list by gender within 10-year age strata is proportional to the corresponding population distributions. Selected subjects are mailed an invitation to participate, and, if they do not respond, up to 7 telephone attempts are made at different times on various days of the week. If telephone contact is unsuccessful, 2 more letters are mailed. Subjects not reached are replaced using the same selection protocol. Subjects who refuse to participate are not replaced. Each participant receives several self-administered, standardized questionnaires covering the risk factors for the major lifestyle chronic diseases, socio-demographic characteristics, educational and occupational histories, and reproductive history for women. The 1999–2009 mean participation rate was 60% (range: 55%–65%).

### Measurements

Each participant brings along their filled-in questionnaires, which are checked for correct completion by trained interviewers (a full visit can be watched on http://epidemiologiepopulation.hug-ge.ch/video_busSante.html). In a temperature-controlled room, body weight is measured with the subject lightly dressed without shoes using a medical scale (precision 0.5 kg), and standing height is measured using a medical gauge (precision 1 cm). BP is measured thrice in the sitting position on the right arm after at least 10 minutes rest using a standard protocol. Between 1999 and 2003, BP was measured using a manual mercury sphygmomanometer. Since 2004, BP was measured using a validated automated oscillometric sphygmomanometer (Omron® HEM-907, Matsusaka, Japan).

### Prevalence, Unawareness, Treatment and Control of Hypertension (Figure S1)

Hypertension was defined as mean systolic and/or diastolic BP≥140/90 mmHg or self-reported hypertension or presence of anti-hypertensive medication. We considered as unaware participants with high BP values who responded negatively to the question “Have you ever been told that you had high BP?” Self-reported anti-hypertensive medication in participants determined treated hypertension. Uncontrolled hypertension was defined as a mean systolic and/or diastolic BP≥140/90 mmHg based on the Seventh Joint National Committee (JNCVII) on Detection, Evaluation, and Treatment of High Blood Pressure [10].

### Physical Activity and Alcohol Consumption

Physical activity levels were quantified using a physical activity frequency questionnaire (PAFQ), developed in the Geneva general adult population and validated using heart rate monitoring [18]. Sedentarity was defined as 10 or less percent of total daily energy expenditure (EE) (kcal/day) spent in activities demanding ≥4 MET as suggested elsewhere [19].

Alcohol consumption (kcal/day) was estimated using a validated food frequency questionnaire [20], [21]. Alcohol consumption was categorized into tertiles (lower, middle, upper tertiles).

### Education, Citizenship, Medical History and Comorbidities

Self-reported information on education was categorized as high (Maturity/Baccalaureat or university) and low (Elementary school or apprenticeship). Citizenships were categorized as Swiss and non-Swiss according to self-reported nationality. Self-reported information on lifestyle, medical history and comorbidities included smoking status (never smokers, ex-smokers, and current smokers), history of myocardial infarction, diabetes, and hypercholesterolemia. Diabetes and hypercholesterolemia were defined as follows: positive responses to the questions: “Have you ever been told that you had diabetes/high cholesterol?” and “If so, are you taking a drug for it?”.

### Socioeconomic Status

Since 2005, socioeconomic information is collected in the ‘Bus Santé’ study. Self-reported monthly household income is collected using the following ranges: <3′000 CHF; 3′000–4′999 CHF; 5′000–6′999 CHF; 7′000–9′499 CHF; 9′500–13′000 CHF; >13′000 CHF (Swiss Francs CHF≈1.1 US$≈0.80€ as on January 2012). Job position information was categorized as non-manual, manager or independent**;** non-manual, employed; manual, independent; manual, employed; women/man-at-home; retired or jobless.

### Statistical Analyses

Statistical analyses were performed using Stata 11.0 (Stata Corp, College Station, USA). Continuous variables were expressed as mean ± standard deviation (SD). Categorical variables were expressed as number of subjects and percentage. To determine whether the prevalence, unawareness, treatment and control of hypertension changed between 1999 and 2009, yearly data were used and trend test performed. To test the associations between potential determinants (e.g. smoking status) and study outcomes (e.g. untreated hypertension), period 1999–2003 and 2004–2009 were used. The periods were so defined because of the publication of the last Joint National Committee (JNCVII) on Detection, Evaluation, and Treatment of High Blood Pressure report (2003) [10], and because of the change in BP measurement (1999–2003: mercury sphygmomanometer; 2004–2009: automated oscillometric sphygmomanometer). To adjust for the age (a major risk factor of hypertension) structure between survey periods, we reported age-standardized prevalence, using the 2010 Geneva Census population. We determined the associations of characteristics with hypertension, hypertension unawareness, untreated and uncontrolled hypertension using multiple logistic regressions.

In the years 2005 through 2008 the annual average number of participants was lower than the other years because another cohort study was conducted relying on the same infrastructure. This is why sample size is smaller for the 2004–2009 than for the 1999–2003 period. Trends in prevalence of hypertension, hypertension unawareness, untreated and uncontrolled hypertension were further determined by income level in a sub-population taking into account information on job position status.

### Ethics

The ‘Bus Santé’ study complied with the Declaration of Helsinki and was approved by the Institutional Ethics Committee of the University of Geneva (Paul Bovier, Bernard Baertschi, Patrick Bovier, Jacquline Bursik, Béat Stoll, Marinette Ummel). All participants gave written informed consent.

## Results

### Population Characteristics

A total of 9,215 subjects were included in the analyses (50% women). Overall mean age was 51.5 yrs (SD, 10.8). There were 4,402 subjects between 35–49 yrs (47.8%), 3,462 between 50–64 yrs (37.6%) and 1,351 between 65–74 yrs (14.7%) (**Table 1**). Forty-five percent of the subjects were never-smokers, 30% were current smokers and 25% ex-smokers. Average BMI was 25.1 (SD 4.2) kg/m^2^. Twelve percent had a BMI of 30 kg/m^2^ or more (obesity). Twenty-six percent had hypercholesterolemia, 6.1% diabetes, 1.9% a history of myocardial infarction. About half of the participants reported high education level.

**Table 1:** Participants’ characterisitics, values are Mean (SD) or N (%).

	ALL	Period 1	Period 2	
	1999–2009 (N = 9,215)	1999–2003 (N = 6,020)	2004–2009 (N = 3,195)	P value
Age, mean, (yrs)	51.5 (10.8)	51.5 (10.8)	51.5 (10.9)	0.88
Age group				
35–49yrs	4,402 (47.8)	2,874 (47.7)	1,528 (47.8)	0.89
50–64yrs	3,462 (37.6)	2,270 (37.7)	1,192 (37.3)	
65–74yrs	1,351 (14.7)	876 (14.6)	475 (14.9)	
Female	4,605 (50.0)	2,977 (49.5)	3043 (50.5)	0.17
Smoking status				
Never smokers	4,140 (44.9)	2,681 (44.5)	1,459 (45.7)	**0.001**
Current smokers	2,739 (29.7)	1,741 (28.9)	998 (31.2)	
Ex-smokers	2,336 (25.3)	1,598 (26.5)	738 (23.1)	
BMI, mean, (kg/m^2^)	25.1 (4.2)	25.1 (4.2)	25.0 (4.2)	0.26
BMI, categories,				
BMI <25 kg/m^2^	4,852 (52.7)	3,160 (52.5)	1,692 (53.0)	0.91
BMI 25–29.9 kg/m^2^	3,273 (35.5)	2,147 (35.7)	1,126 (35.2)	
BMI ≥30 kg/m^2^	1'090 (11.8)	713 (11.8)	377 (11.8)	
Diabetes	563 (6.1)	343 (5.7)	220 (6.9)	**0.02**
Hypercholesterolemia	2,408 (26.1)	1,525 (25.3)	883 (27.6)	**<0.0001**
Myocardial infarction history	177 (1.9)	123 (2.0)	54 (1.7)	0.24
SBP, mm Hg, mean	125.9 (19.4)	126.1 (19.7)	125.5 (18.8)	0.13
DBP, mm Hg, mean	78.0 (11.2)	79.1 (11.0)	76.0 (11.2)	**<0.0001**
HTN stages				**<0.0001**
S/DBP≥160/≥100 mm Hg	716 (7.8)	507 (8.4)	209 (6.5)	
S/DBP 140–159/90–99 mm Hg	1′908 (20.7)	1,369 (22.7)	539 (16.9)	
S/DBP 120–139/80–89 mm Hg	3′510 (38.1)	2,328 (38.7)	1,182 (37.0)	
S/DBP <120/<80 mm Hg	3′081 (33.4)	1,816 (30.2)	1,265 (39.6)	
Education level				
Low = Elementary school or apprenticeship	4,429 (48.1)	2,837 (47.1)	1,592 (49.8)	**0.01**
High = Maturity/baccalaureat or university	4,786 (51.9)	3,183 (52.9	1,603 (50.2)	
Swiss citizenship	4,916 (54.2)	3,321 (55.3)	1,595 (52.2)	**0.007**
Sedentarity	5,863 (63.6)	3,836 (63.7)	2,027 (63.4)	0.79
Alcohol consumption				**<0.0001**
Lower tertile	3,086 (33.5)	1,963 (32.6)	1,123 (35.1)	
Middle tertile	3,073 (33.4)	1,963 (32.6)	1,110 (34.7)	
Upper tertile	3,056 (33.2)	2,094 (34.8)	962 (30.19)	

BMI, body mass index; SBP, systolic blood pressure; DBP, diastolic blood pressure; HTN, hypertension.

Compared to participants, non-participants were more likely to be women (52.2% vs 50.0%, p value <0.001), older (52.7 years (SD 11.1) vs 51.5 (SD 10.8), p value <0.001) and to have the Swiss citizenship (62.9% vs 54.2%, p value <0.001). There was no difference with respect to smoking status (47.0% vs 44.9% were never smokers, 29.2% vs 29.7% were current smokers, and 23.9% vs 25.3% were ex-smokers, p value = 0.06).

The mean systolic and diastolic BP were 125.9 mmHg (19.4) and 78.0 mmHg (11.2), respectively. The percentages of participants by BP categories were as follow: systolic and diastolic BP<120/<80, 33.4%; systolic or diastolic BP 120–139/80–89, 38.1%; systolic or diastolic BP 140–159/90–99, 20.7%; and systolic or diastolic BP≥160/≥100, 7.8%.

The number of participants were 6,020 (65.3%) and 3,195 (34.7%) in the 1999–2003 and 2004–2009 survey period, respectively. The prevalence of smoking status, diabetes, hypercholesterolemia, education level, Swiss citizenship, and alcohol consumption differed significantly between the two periods. The mean diastolic BP was lower in the second period than the first period (79.1 vs 76.0, p<0.05), and the overall distribution of the grades or stages of hypertension differed between the two periods.

### Trends in Hypertension, Unawareness, Untreated and Uncontrolled Hypertension Prevalences

**Figure 1:**
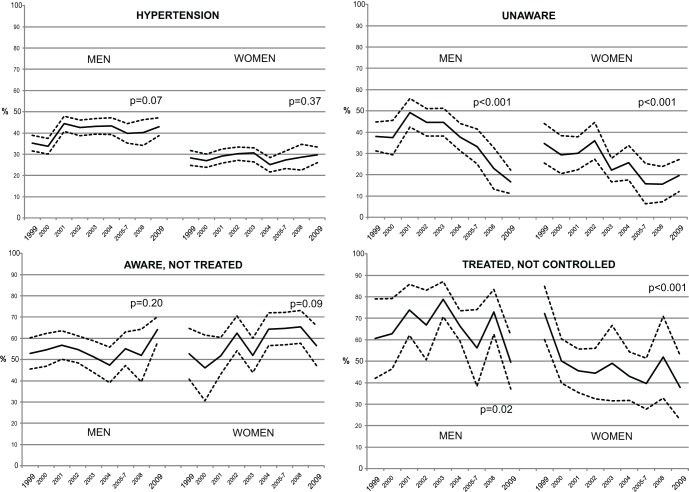
Age-standardized prevalences of hypertension, unawareness, untreated, and uncontrolled hypertension, by gender and survey year.

The overall age-standardized prevalence of hypertension was 34.4%. This prevalence remained stable between 1999–2009 (**Figure 1** **, Tables S1 and S2**) in both men and women. The prevalence of hypertension unawareness decreased between 1999 and 2009, in men and in women specifically (p<0.001). In 1999, 35.90% of hypertensive participants were unaware of having hypertension, whereas 17.7% of hypertensive participants were unaware of having hypertension in 2009., The increasing tendancy of the prevalence of untreated hypertension observed in men and in women specifically was only statistically significant (from 53.9% to 61.8%, p = 0.04) when men and women participants were combined and thus power increased. The prevalence of uncontrolled hypertension decreased between 1999 and 2009 (p<0.05); from 62.2% to 40.6% in men and women combined, from 60.6% to 49.6% in men, and from 72.2% to 37.8% in women.

### Determinants of Hypertension

Multivariate associations of characteristics with hypertension are reported by periods in **Table 2**. In the 1999–2003 period, male gender, sedentarity, obesity, hypercholesterolemia, diabetes, older age, and alcohol consumption were all positively associated with the risk of hypertension. Compared to never and ex-smokers, current smokers were less likely to have hypertension.

**Table 2:** Multivariate associations (odds ratios, 95%CI, p values) of characteristics with hypertension, hypertension unawareness, untreated hypertension and uncontrolled hypertension, for the period 1999–2003 and 2004–2009.

	Hypertension		Hypertension unawareness		Untreated hypertension		Uncontrolled hypertension	
Period	1999–2003	2004–2009	1999–2003	2004–2009	1999–2003	2004–2009	1999–2003	2004–2009
Current smoker *vs* never/exsmoker	0.82* (0.72–0.94)p = 0.005	0.75* (0.30–0.92) p = 0.007	1.27*(1.02–1.59) p = 0.031	1.17 (0.80–1.70)p = 0.419	1.07 (0.78–1.47)p = 0.668	1.29 (0.83–2.00)p = 0.255	0.99 (0.65–1.49)p = 0.949	0.96 (0.55–1.68)p = 0.894
Male *vs* female	1.64* (1.45–1.88)p<0.001	1.97* (1.64–2.36) p<0.001	1.56* (1.27–1.92) p<0.001	1.32 (0.96–1.83)p = 0.091	1.00 (0.74–1.34)p = 0.982	0.78 (0.55–1.12)p = 0.185	1.49* (1.03–2.17)p = 0.256	1.65* (1.08–2.50)p = 0.022
Swiss *vs* non-Swiss	1.00 (0.88–1.12)p = 0.938	0.92 (0.78–1.10) p = 0.359	0.84 (0.70–1.01) p = 0.064	0.93 (0.70–1.25)p = 0.648	0.91 (0.70–1.19)p = 0.495	1.36 (0.98–1.89)p = 0.067	0.83 (0.59–1.15)p = 0.256	0.75 (0.51–1.12)p = 0.160
Sedentarity	1.22* (1.08–1.39)p = 0.002	1.05 (0.88–1.26) p = 0.574	1.05 (0.86–1.28) p = 0.660	0.87 (0.64–1.19)p = 0.386	0.72* (0.54–0.95) p = 0.020	0.91 (0.64–1.30)p = 0.623	1.56* (1.08–2.24)p = 0.017	1.09 (0.71–1.69)p = 0.689
Obesity	3.19* (2.67–3.81)p<0.001	2.84* (2.21–3.66) p<0.001	0.73* (0.58–0.92) p = 0.008	0.65* (0.44–0.97)p = 0.035	0.83 (0.61–1.14)p = 0.252	0.77 (0.51–1.15)p = 0.199	1.31 (0.89–1.94)p = 0.167	0.92 (0.58–1.47)p = 0.740
Hypercholesterolemia	1.09*(1.03–1.14) p = 0.001	1.13* (1.01–1.27) p = 0.001	1.31*(1.20–1.44) p<0.001	1.17 (0.96–1.42)p = 0.127	0.59* (0.45–0.77)p<0.001	0.75 (0.54–1.06)p = 0.101	0.96 (0.69–1.34)p = 0.825	0.90 (0.60–1.35)p = 0.613
Diabetes	1.86* (1.44–2.39)p<0.001	2.01* (1.44–2.80) p<0.001	0.36* (0.25–0.51) p<0.001	0.41* (0.26–0.70)p = 0.001	0.69 (0.45–1.05)p = 0.085	0.71 (0.44–1.16)p = 0.174	0.96 (0.61–1.52)p = 0.874	1.15 (0.69–1.94)p = 0.592
50–64y *vs* 35–49y	2.94* (2.57–3.35)p<0.001	3.18* (2.63–3.85) p<0.001	1.14 (0.91–1.43) p = 0.247	1.02 (0.71–1.47)p = 0.924	0.23* (0.17–0.31)p<0.001	0.14* (0.09–0.22)p<0.001	1.32 (0.81–2.16)p = 0.268	0.99 (0.46–2.13)p = 0.976
65–74 *vs* 35–49y	7.64* (6.39–9.14)p<0.001	8.60* (6.66–11.10) p<0.001	1.56* (1.21–2.02) p<0.001	1.00 (0.66–1.50)p = 0.982	0.06*(0.04–0.10)p<0.001	0.06*(0.03–0.09)p<0.001	1.97 (1.17–3.32)p = 0.011	1.03 (0.47–2.26)p = 0.936
Alcohol consumptionmiddle *vs* lower tertile	0.97 (0.83–1.3)p = 0.699	1.05 (0.85–1.30) p = 0.630	0.93 (0.73–1.18) p = 0.546	1.04 (0.71–1.51)p = 0.854	1.07 (0.76–1.50)p = 0.696	0.83 (0.54–1.25)p = 0.368	1.03 (0.68–1.56)p = 0.894	0.61 (0.37–1.00)p = 0.050
Alcohol consumptionupper *vs* lower tertile	1.18* (1.01–1.38)p = 0.033	1.22 (0.98–1.52) p = 0.074	1.05 (0.83–1.34) p = 0.668	1.16 (0.79–1.70)p = 0.439	0.95 (0.67–1.34)p = 0.781	1.00 (0.66–1.54)p = 0.985	1.16 (0.76–1.77)p = 0.496	1.04 (0.63–1.73)p = 0.877
Education High level vs low level	0.90 (0.80–1.02)p = 0.091	0.72* (0.61–0.86) p<0.001	1.08 (0.90–1.30) p = 0.402	0.85 (0.63–1.14)p = 0.280	1.45* (1.12–1.88)p = 0.005	1.42* (1.02–1.99)p = 0.039	1.00 (0.72–1.39)p = 0.988	0.79 (0.53–1.18)p = 0.244

Odds ratios with p values <0.05 are marked with an asterix.

Similar associations were found in the 2004–2009 with the exception of sedentarity, alcohol consumption, and education level. The first two were not positively associated with hypertension in the second survey period. Of note, high education was significantly negatively associated with hypertension in the second survey period.

### Determinants of Hypertension Unawareness

In the 1999–2003 period, current smokers were more likely to be unaware of having hypertension compared to never and ex-smokers. Male gender, hypercholesterolemia, older age were positively associated with the risk of hypertension unawareness. Participants with BMI ≥30 kg/m^2^ (and diabetes were less likely to be unaware of having hypertension. In the 2004–2009 period, only obesity and diabetes remained independently associated with hypertension unawareness.

### Determinants of Untreated Hypertension

In the first survey period, sedentarity, hypercholesterolemia, and older age were associated with a lower risk of untreated hypertension. Participants with high education were more likely to have untreated hypertension than participants reporting low level of education. Older age and education level remained associated with untreated hypertension in the 2004–2009 period.

### Determinants of Uncontrolled Hypertension

Male gender and sedentarity were associated with increased risk of uncontrolled hypertension in the first survey period, while only male gender remained associated with uncontrolled hypertension in the second survey period.

### Prevalence of Hypertension, Unawareness, Untreated and Uncontrolled Hypertension, by Monthly Household Income and Job Position

Information on monthly household income and job position collected since 2005 was available for 2,024 (22.0%) participants whose main characteristics are presented in **Table S3**. The age-standardized prevalence of hypertension, unawareness, untreated and uncontrolled hypertension varied with job position (**Table S4**). To adjust for the effect of job position, trends with household monthly income were further adjusted for job position. After full adjustment, no clear trends were found.

### Prevalences in Mutually Exclusive Groups

Figure S2 illustrated the prevalences of the four mutually exclusive groups among participants with hypertension: unaware; aware, not treated; aware, treated, not controlled; aware, treated, and controlled. The prevalences of these mutally exclusive groups for the period 1999–2003 and 2004–2009 were respectively 38.6% (809/2095) and 24.9% (272/1094), 33.2% (695/2095) and 43.8% (479/1094), 17.0% (357/2095) and 18.9% (207/1094), 11.2% (234/2095) and 12.4% (136/1094).

## Discussion

Hypertension is the most prevalent cardiovascular disorder in high-income countries, where it affects 20% to 50% of the adult population [2], [22]. In a representative population of adults in Geneva, the 2004–2009 age-adjusted prevalence of hypertension were 41.7% and 27.3% in men and women, respectively. These prevalences are higher than the ones reported in the United States, but similar to what is found in most European countries [23]–[28]. This is in line with previous reports showing that hypertension prevalences are higher in Europe than in North America. Wolf Maier et al. found that hypertension prevalence was 28% in the North American countries and 44% in 6 European countries [6].

Our analyses showed no significant changes in hypertension prevalence in adults from 1999 to 2009. A 1993–2000 analysis conducted in the same source population reported, a decline in hypertension prevalence in men and women [29]. It was suggested that the decline could be attributed to dietary changes such as changes in salt intake [29]. Salt intake in the Geneva population has however remained particularly high and stable between 1993 and 2004 [30]. Our results showing an absence of further decline in hypertension prevalence in recent years are in line with other major surveys. The most recent NHANES survey reported an increase in hypertension prevalence followed by a stable prevalence around 29% in the 2007–2008 period [4]. Data from the MONICA studies reported different trends in Belgium, Finland and Germany; a decline, a stable, and an increase prevalence of hypertension, respectively [2]. The Health Survey for England conducted in 1994 and 1998 reported similar prevalence of hypertension (about 37%) [2]. Thus, much needs to be done to decrease the burden of hypertension in the canton of Geneva and population-based strategies, such as reduction of sodium intake, are of utmost importance.

Hypertension awareness improved between 1999 and2009 in the Geneva population. Similar favorable trends have been reported in other, yet scarce, longitudinal reports on hypertension awareness [5], [12], [31]–[33]. In the last period (2004–2009), about 25% of the participants were unaware of having hypertension. This is lower than most of the estimates reported in other European cross-sectional studies performed after 2002 [25], [26], [28], [34], [35]. Current smoking, male gender, hypercholesterolemia, and older age were associated with hypertension unawareness. Smoking has been consistently described as a barrier to preventive medicine such as screening, but association between smoking and hypertension unawareness has rarely been looked at or found [7], [14], [16], [36]. In a representative sample of the Chinese population (n = 15 838), higher unawareness was also found, with the same magnitude as in our study population (OR = 1.27) [15]. Men with hypertension were less frequently aware than women, a finding consistent with the literature [4], [7], [28], [35], [37]. It has been suggested that the gender difference in awareness is, in part, due to the more frequent lifetime contact of women with the medical staff.

Elevated BP is a risk factor for coronary heart disease, heart failure, stroke, peripheral arterial disease, and renal failure [38]–[41]. BP control is adequate when systolic and diastolic BPs are <140 mm Hg and <90 mm Hg, respectively [42], [43]. Prevalence of uncontrolled BP in hypertensive patients varied from 10% to 65% across studies [2], [24], [44]–[51]. We found that hypertension was detected but often untreated in the Geneva population. In the last survey period, the prevalence of untreated hypertension was 58% in the canton of Geneva, which lies in the upper range of European’s estimates [25]–[27], [35]. In France, the neighboring country of Geneva, the prevalence of untreated hypertension was only 20% in the 2005–2007 MONA LISA [35]. Our data suggested that untreated hypertension has been stable since 1999. The substantial decrease in hypertension unawareness between 1999–2003 and 2004–2009 was therefore not paralleled by a concomitant absolute increase in hypertension treatment. As a consequence, a larger proportion of all hypertensive participants were aware but not treated in 2004–2009 (43.7%) compared to 1999–2003 (33.1%) (**Figure S2**). The observed gain in hypertension awareness is therefore unlikely to translate into public health benefit if no action is undertaken to control BP. We found that BP was not controlled in half of hypertensive treated subjects, which corresponds to the control rate reported in 2009 in a Swiss city population-based study (48%) [7].

Hypertension treatment initiation and intensification once hypertension is detected are challenged by both patient- and physician-related factors, which may vary across regions [52]. Outclinic patients with inadequately controlled hypertension seemed to be less likely to receive a medication increase in the United States than in European countries [53]. In Switzerland, information on reasons for untreated and/or uncontrolled hypertension is limited. In a family practice based, open intervention survey, physicians-related reasons for uncontrolled BP were to believe that baseline BP dictates the target, that a clear improvement in BP might be sufficient and that the full drug effect may take up to 4 months or more to be achieved [54]. Here, we provide information on patients-related characteristics of untreated and uncontrolled hypertension. This needs to be completed by additional patient- and physician-based studies conducted in Switzerland.

After adjustment, participants with high education level were at increased risk of untreated hypertension than participants with lower level of education. The associations between education and hypertension awareness, treatment and control are inconsistent. Some reports showed an inverse association or no association between education level and hypertension awareness, treatment or control [16]. In line with our results, untreated hypertension was positively associated with high education level in four Scottish MONICA cross-sectional surveys (1986, 1989, 1992 and 1995) [55]. Reason for this unexpected, yet replicated, association is not clear. It has been suggested that people with high education level might think they are able to manage their BP and might therefore not follow their GP’s advice. Because such interventions are highly dependent on educational level and motivation, we propose that, conversely, nonpharmacological lifestyle interventions might be more often presented by GP’s to better educated subjects.

Our results have to be interpreted within the context of the Swiss health care system. Health insurance is compulsory for all citizens of Switzerland (7 millions) and insurance premiums are paid independently of earnings [56], [57]. Subsidies are paid for citizens with low income. Health insurance covers the costs of medical treatment and hospitalisation of the insured. Everyone pays part of the cost of treatment through 1) an annual flat deductible, called the ‘franchise’, which ranges from CHF 300 to a maximum of CHF 2,500 (1CHF ≈ 1$ ≈ 1.35€), at the insured person’s choice (premiums are adjusted accordingly); and 2) a 10% deductible of the costs up to a stop-loss amount of CHF 700 per year. Some 40% of the Swiss population chooses to top up their insurance coverage with private health insurance, which offers a wider choice of treatments and health care professionals, or more comfortable accommodation during a hospital stay. In contrast to basic insurance, insurers may refuse applicants for private insurance or only accept them subject to conditions. While all basic insurances will cover medical (primary care and specialist) consultations as well as anti-hypertensive treatment, the insured still pays 10% of the cost (i.e. out-of pocket participation). Switzerland has the highest out of pocket participation within countries of the Organisation for Economic Co-operation and Development [58]. Thus, even though the Swiss health care insurance coverage is universal, patient’s financial ressources may still influence the use of medical services and treatment. Yet, we found no independent associations between monthly household income – a proxy of an individual financial resources – and hypertension, awareness, treatment, or control.

### Strengths and Limitations

When interpreting the findings of this study, one has to keep in mind its limitations. Non participants slightly differed from participants with respect to sex, age and Swiss citizenship. Given that our results suggested that age, smoking status and gender are significant predictors of study outcomes, these differences may somewhat limit the generalisability of our findings. Several informations were determined by the use of questionnaires. By nature of its reliance on self-reported data, this is a source of possible bias. Similarly to other large population-based studies, white coat effect, white coat hypertension, or masked hypertension could not be determined in this analysis. BP was measured with two different methods; mercury sphygmanometer (1999–2003) and semi-automatic oscillometer (2004–2009). Although semi-automatic oscillometer are calibrated with mercury sphygmanometer, we cannot exclude that some of the differences observed between the two periods are attributable to the method of BP measurement. Yet, it is also possible that the 2003 antihypertension guidelines (e.g. the 2003 Seventh Joint National Committee (JNCVII) [10], the 2003 World Health Organization (WHO)/International Society of Hypertension (ISH) and European statement on management of hypertension [11]) contributed to improve the levels of awareness and adherence of the medical staff to hypertension screening and control. The study which was conducted concomitantly to the Bus Santé study between 2005 and 2008 was a follow-up study involving different independent participants than the Bus Santé study. During this period, only a smaller number of subjects from the Geneva population were randomly selected and invited to participate to the Bus Santé study. The study protocole and procedures remained identical. Yet, we cannot exclude some interference between the two studies. The strengths of this study are the secular comparison of prevalences using the same definition, in the same source population, using the same number of BP readings. In addition, the large number of included subjects (>9′000), the recruitment strategy of the participants (representative sampling and low attrition), the time window (ten years with yearly data), and the inclusion of objective measures strengthen the findings.

In conclusion, in a representative sample of the canton of Geneva, Switzerland, the prevalence of hypertension remained stable between 1999–2003 and 2004–2009. Population-based primary prevention measures are needed to decrease the burden of hypertension in this region. While favorable trends in hypertension unawareness and uncontrolled hypertension occurred during this period, about half of hypertensive subjects were not treated or had uncontrolled high BP in the latest 2004–2009 survey period. Factors associated with untreated and uncontrolled hypertension in our analyses could guide the implementation of targeted interventions aimed at reducing these rates.

## Supporting Information

Figure S1 **Flow chart of age-standardized prevalences of hypertension, unawareness, untreated, and uncontrolled hypertension, by survey period.**
(EPS)

Figure S2 **Age-standardized prevalences of the four mutually exclusive categories of participants with hypertension, by survey period.**
(EPS)

Table S1 **Age-standardized prevalences (95%CI) of hypertension, hypertension unawarenness, untreated and uncontrolled hypertension, by survey year and gender (N = 9,215).**
(DOCX)

Table S2 **Crude numbers of participants by hypertension, hypertension unawarenness, untreated and uncontrolled hypertension groups, by survey year and gender (N = 9,215).**
(DOCX)

Table S3 **Patients’ characteristics among subjects with socioeconomic information (N = 2,024).**
(DOCX)

Table S4 **Age-standardized prevalence (95%CI) of hypertension, hypertension unaeareness, untreated hypertension, and uncontrolled hypertension by monthly household income and job position, and adjusted trends with income (N = 2,024).**
(DOCX)
