# Supplementary material for: 1999–2009 Trends in Prevalence, Unawareness, Treatment and Control of Hypertension in Geneva, Switzerland
Source: PLoS One. 2012 Jun 27;7(6):e39877. doi: 10.1371/journal.pone.0039877 (PMC3384604; doi:10.1371/journal.pone.0039877)
Supplement: Table S1 — Age-standardized prevalences (95%CI) of hypertension, hypertension unawarenness, untreated and uncontrolled hypertension, by survey year and gender (N = 9,215). (DOCX) [file pone.0039877.s003.docx]

**Table S1. Age-standardized prevalences (95%CI) of hypertension, hypertension unawarenness, untreated and uncontrolled hypertension, by survey year and gender (N=9,215)**

| **Men and women** | **ALL (N=9215)** | **1999 (N=1154)** | **2000 (N=1142)** | **2001 (N=1235)** | **2002 (N=1257)** | **2003 (N=1232)** | **2004 (N=1038)** | **2005-7 (N=739)** | **2008 (N=415)** | **2009 (N=1003)** | **P value for trends** |
| --- | --- | --- | --- | --- | --- | --- | --- | --- | --- | --- | --- |
| Hypertensive, % | 34.4 | 31.5 | 30.2 | 36.9 | 36.3 | 36.4 | 33.8 | 33.5 | 34.4 | 36.1 | 0.07 |
| 95%CI | (33.5-35.3) | (29.0-34.1) | (27.8-32.6) | (34.4-39.4) | (33.9-38.8) | (33.8-38.9) | (31.2-36.5) | (30.3-36.6) | (30.0-38.7) | (33.3-38.9) |  |
| Unaware, % | 33.8 | 35.9 | 34.7 | 42.7 | 41.5 | 36.7 | 34.8 | 26.5 | 20.3 | 17.7 | **<0.001** |
| 95%CI | (31.9-35.7) | (30.4-41.5) | (28.3-41.2) | (37.3-48.1) | (36.4-46.7) | (31.7-41.7) | (29.4-40.3) | (19.9-33.0) | (13.2-27.4) | (13.3-22.2) |  |
| Aware, not treated, % | 55.6 | 53.9 | 51.7 | 54.8 | 58.1 | 51.6 | 53.8 | 58.4 | 59.8 | 61.8 | **0.040** |
| 95%CI | (53.6-57.6) | (47.8-60.1) | (44.1-59.2) | (49.5-60.2) | (52.9-63.2) | (46.1-57.2) | (47.3-60.3) | (52.4-64.4) | (51.7-67.9) | (56.7-66.9) |  |
| Treated, not controlled, % | 56.7 | 62.2 | 52.0 | 54.9 | 52.4 | 64.2 | 61.4 | 51.6 | 71.7 | 40.6 | **0.020** |
| 95%CI | (51.5-61.9) | (46.9-77.4) | (41.4-62.6) | (45.0-64.8) | (39.6-65.1) | (52.7-75.7) | (54.4-68.5) | (37.1-66.2) | (63.5-80.0) | (29.1-52.0) |  |
| **Men only** | **ALL (N=4610)** | **1999 (N=601)** | **2000 (N=557)** | **2001 (N=632)** | **2002 (N=639)** | **2003 (N=614)** | **2004 (N=507)** | **2005-7 (N=372)** | **2008 (N=210)** | **2009 (N=478)** | **P value for trends** |
| Hypertensive, % | 40.5 | 35.3 | 33.7 | 44.5 | 42.6 | 43.2 | 43.3 | 39.8 | 40.2 | 43.0 | 0.07 |
| 95%CI | (39.2-41.8) | (31.6-39.0) | (30.1-37.4) | (40.8-48.1) | (38.8-46.1) | (39.5-46.9) | (39.3-47.2) | (35.3-44.4) | (34.1-46.3) | (38.8-47.2) |  |
| Unaware, % | 37.9 | 38.0 | 37.4 | 49.2 | 44.6 | 44.7 | 37.6 | 33.3 | 23.0 | 16.6 | **<0.001** |
| 95%CI | (35.5-40.4) | (31.2-44.8) | (29.3-45.5) | (42.5-55.9) | (38.1-51.0) | (38.2-51.2) | (31.1-44.1) | (25.1-41.4) | (13.2-32.7) | (11.1-22.1) |  |
| Aware, not treated, % | 54.4 | 52.9 | 54.6 | 56.8 | 54.8 | 51.3 | 47.4 | 55.1 | 51.9 | 64.1 | 0.20 |
| 95%CI | (51.8-57.0) | (45.5-60.3) | (46.8-62.3) | (50.2-63.5) | (48.5-61.1) | (43.8-58.8) | (39.1-55.8) | (47.1-63.1) | (39.5-64.3) | (58.1-70.1) |  |
| Treated, not controlled, % | 61.5 | 60.6 | 62.8 | 73.9 | 66.9 | 78.9 | 66.4 | 56.2 | 73.0 | 49.6 | **0.020** |
| 95%CI | (54.8-68.2) | (42.1-79.1) | (46.4-79.2) | (62.1-85.8) | (50.7-83.0) | (70.7-87.1) | (59.3-73.4) | (38.3-74.1) | (62.7-83.4) | (37.1-62.1) |  |
| **Women, only** | **ALL (N=4605)** | **1999 (N=553)** | **2000 (N=585)** | **2001 (N=603)** | **2002 (N=618)** | **2003 (N=618)** | **2004 (N=531)** | **2005-7 (N=367)** | **2008 (N=205)** | **2009 (N=525)** | **P value for trends** |
| Hypertensive, % | 28.3 | 28.2 | 27.0 | 29.2 | 30.3 | 30.7 | 25.1 | 27.4 | 28.6 | 29.7 | 0.37 |
| 95%CI | (27.1-29.5) | (24.7-31.8) | (23.8-30.1) | (25.9-32.5) | (27.1-33.4) | (26.4-33.1) | (21.7-28.4) | (23.3-31.6) | (22.5-34.7) | (26.1-33.4) |  |
| Unaware, % | 26.6 | 34.7 | 29.4 | 30.1 | 36.0 | 22.1 | 25.6 | 15.8 | 15.6 | 19.6 | **<0.001** |
| 95%CI | (23.7-29.4) | (25.4-44.1) | (20.5-38.3) | (22.3-37.9) | (27.3-44.7) | (16.6-27.7) | (17.5-33.7) | (6.3-25.3) | (7.3-23.9) | (12.0-27.2) |  |
| Aware, not treated, % | 56.9 | 52.7 | 46.0 | 51.8 | 62.4 | 52.0 | 64.3 | 64.6 | 65.4 | 56.5 | 0.09 |
| 95%CI | (53.7-60.0) | (40.8-64.6) | (30.6-61.5) | (43.1-60.5) | (54.2-70.5) | (43.8-60.1) | (56.5-72.1) | (57.0-72.2) | (57.7-73.2) | (47.1-66.0) |  |
| Treated, not controlled, % | 49.2 | 72.2 | 50.2 | 45.6 | 44.3 | 49.1 | 43.0 | 39.6 | 51.9 | 37.8 | **<0.001** |
| 95%CI | (41.1-57.3) | (60.0-84.9) | (39.9-60.4) | (35.5-55.7) | (32.4-56.1) | (31.5-66.7) | (31.7-54.4) | (27.7-51.5) | (32.9-70.9) | (22.8-52.8) |  |
