# Supplementary material for: 1999–2009 Trends in Prevalence, Unawareness, Treatment and Control of Hypertension in Geneva, Switzerland
Source: PLoS One. 2012 Jun 27;7(6):e39877. doi: 10.1371/journal.pone.0039877 (PMC3384604; doi:10.1371/journal.pone.0039877)
Supplement: Table S2 — Crude numbers of participants by hypertension, hypertension unawarenness, untreated and uncontrolled hypertension groups, by survey year and gender (N = 9,215). (DOCX) [file pone.0039877.s004.docx]

**Table S2. Crude numbers of participants by hypertension, hypertension unawarenness, untreated and uncontrolled hypertension groups, by survey year and gender (N=9,215)**

| **Men and women** | **ALL (N=9215)** | **1999 (N=1154)** | **2000 (N=1142)** | **2001 (N=1235)** | **2002 (N=1257)** | **2003 (N=1232)** | **2004 (N=1038)** | **2005-7 (N=739)** | **2008 (N=415)** | **2009 (N=1003)** |
| --- | --- | --- | --- | --- | --- | --- | --- | --- | --- | --- |
| Hypertensive | 3199/9215 | 359/1154 | 348/1142 | 482/1235 | 455/1257 | 451/1232 | 344/1038 | 238/739 | 146/415 | 366/1003 |
| Unaware | 1105/3199 | 135/359 | 133/348 | 214/482 | 187/455 | 166/451 | 119/344 | 57/248 | 33/146 | 61/366 |
| Aware, not treated | 890/2094 | 98/224 | 85/215 | 104/164 | 120/268 | 114/25 | 90/225 | 78/191 | 52/113 | 149/305 |
| Treated, not controlled | 769/1204 | 87/126 | 91/130 | 125/164 | 99/148 | 105/171 | 89/135 | 60/113 | 35/61 | 787156 |
| **Men only** | **ALL (N=4610)** | **1999 (N=601)** | **2000 (N=557)** | **2001 (N=632)** | **2002 (N=639)** | **2003 (N=614)** | **2004 (N=507)** | **2005-7 (N=372)** | **2008 (N=210)** | **2009 (N=478)** |
| Hypertensive | 1891/4610 | 214/601 | 193/557 | 295/632 | 271/639 | 265/614 | 213/507 | 146/372 | 86/210 | 208/478 |
| Unaware | 718/1891 | 88/214 | 78/193 | 144/295 | 120/271 | 111/265 | 79/213 | 42/146 | 21/86 | 35/208 |
| Aware, not treated | 498/1173 | 56/126 | 49/115 | 62/151 | 66/151 | 57/154 | 49/134 | 39/104 | 27/65 | 93/173 |
| Treated, not controlled | 455/675 | 48/70 | 44/66 | 70/89 | 63/85 | 67/97 | 60/85 | 38/65 | 23/38 | 42/89 |
| **Women, only** | **ALL (N=4605)** | **1999 (N=553)** | **2000 (N=585)** | **2001 (N=603)** | **2002 (N=618)** | **2003 (N=618)** | **2004 (N=531)** | **2005-7 (N=367)** | **2008 (N=205)** | **2009 (N=525)** |
| Hypertensive | 1308/4605 | 145/553 | 155/585 | 187/603 | 184/618 | 186/618 | 131/531 | 102/367 | 60/205 | 158/525 |
| Unaware | 387/1308 | 47/145 | 55/155 | 70/187 | 67/184 | 55/186 | 40/131 | 15/102 | 12/60 | 26/158 |
| Aware, not treated | 392/921 | 42/98 | 36/100 | 42/117 | 54/117 | 57/131 | 41/91 | 39/87 | 25/48 | 56/132 |
| Treated, not controlled | 314/529 | 39/56 | 47/64 | 55/75 | 36/63 | 38/74 | 29/50 | 22/48 | 12/23 | 36/76 |
