# Supplementary material for: 1999–2009 Trends in Prevalence, Unawareness, Treatment and Control of Hypertension in Geneva, Switzerland
Source: PLoS One. 2012 Jun 27;7(6):e39877. doi: 10.1371/journal.pone.0039877 (PMC3384604; doi:10.1371/journal.pone.0039877)
Supplement: Table S3 — Patients’ characteristics among subjects with socioeconomic information (N = 2,024). (DOCX) [file pone.0039877.s005.docx]

**Table S3 Patients’ characteristics among subjects with socioeconomic information (N=2,024)**

|  | Mean (SD) or N (%) |
| --- | --- |
| Age, mean, (yrs) | 51.6 (11.0) |
| Age group |  |
| 35-49yrs | 952 (47.0) |
| 50-64yrs | 766 (37.9) |
| 65-74yrs | 306 (15.0) |
| Female | 997 (49.3) |
| Never smoker | 903 (44.6) |
| Current smoker | 667 (33.0) |
| Ex-smoker | 454 (22.4) |
| BMI, mean, (kg/m^2^) | 25.0 (4.1) |
| BMI, categories, |  |
| BMI, <25 kg/m^2^ | 1,088 (55.0) |
| BMI 25-29.9 kg/m^2^ | 707 (34.9) |
| BMI ≥30 kg/m^2^ | 229 (11.3) |
| Diabetes | 138 (6.8) |
| Hypercholesterolemia | 583 (28.8) |
| Myocardial infarction history | 35 (1.7) |
| Systolic blood pressure, mmHg, mean | 125.5 (18.1) |
| Diastolic blood pressure, mmHg, mean | 75.5 (11.2) |
| S/DBP ≥160/≥100 | 125 (6.2) |
| S/DBP 140-159/90-99 | 322 (15.9) |
| S/DBP 120-139/80-89 | 786 (38.8) |
| S/DBP <120/ <80 | 791 (39.1) |
| Education level |  |
| Low= Elementary school or apprenticeship | 1,049 (51.8) |
| High= Maturity/baccalaureat or university | 975 (48.2) |
| Swiss citizenship | 1,013 (53.4) |
| Sendarity | 1,272 (63.0) |
| Alcohol consumption |  |
| Lower tertile | 692 (34.2) |
| Middle tertile | 736 (36.4) |
| Upper tertile | 596 (29.5) |
| Monthly household income |  |
| <3,000 CHF | 110 (5.4) |
| 3,000 – 4,999 CHF | 304 (15.0) |
| 5,000 – 6,999 CHF | 376 (18.6) |
| 7,000 – 9,499 CHF | 457 (22.6) |
| 9,500 – 13,000 CHF | 397 (19.6) |
| >13,000 CHF | 380 (18.8) |
| Job position |  |
| Non-manual, manager or independent | 465 (23.0) |
| Non-manual, employed | 510 (25.2) |
| Manual, independent | 115 (5.7) |
| Manual, employed | 338 (16.7) |
| Woman/Man-at-home | 117 (5.8) |
| Retired, jobless, or disability insurance | 479 (23.7) |
