# Supplementary material for: 1999–2009 Trends in Prevalence, Unawareness, Treatment and Control of Hypertension in Geneva, Switzerland
Source: PLoS One. 2012 Jun 27;7(6):e39877. doi: 10.1371/journal.pone.0039877 (PMC3384604; doi:10.1371/journal.pone.0039877)
Supplement: Table S4 — Age-standardized prevalence (95%CI) of hypertension, hypertension unaeareness, untreated hypertension, and uncontrolled hypertension by monthly household income and job position, and adjusted trends with income (N = 2,024). (DOCX) [file pone.0039877.s006.docx]

**Table S4 Age-standardized prevalence (95%CI) of hypertension, hypertension unaeareness, untreated hypertension, and uncontrolled hypertension by monthly household income and job position, and adjusted trends with income (N=2,024)**

|  | **% Hypertensive** | **% Unaware** | **% Aware, not treated** | **%Treated, not controlled** |
| --- | --- | --- | --- | --- |
| ALL (N=2024) | 34.8 (32.9-36.7) | 20.7 (17.3-24.1) | 60.0 (56.4-63.7) | 45.7 (38.1-53.4) |
| *Job position* |  |  |  |  |
| Non-manual, manager or independent (N=465) | 33.1 (29.0-37.3) | 16.0 (10.2-21.8) | 57.0 (49.2-64.8) | 48.2 (31.8-64.5) |
| Non-manual, employed (N=510) | 23.2 (18.5-28.9) | 21.4 (14.0-28.9) | 60.2 (51.3-69.1) | 45.3 (33.7-56.8) |
| Manual, independent (N=115) | 31.9 (24.9-38.9) | 23.1 (11.9-34.3) | 69.2 (60.6-77.8) | 59.0 (47.6-70.4) |
| Manual, employed (N=338) | 35.2 (29.0-41.4) | 23.1 (15.4-30.8) | 65.2 (56.7-73.7) | 41.5 (25.2-57.7) |
| Woman/Man-at-home (N=117) | 29.1 (21.9-36.2) | 19.0 (7.7-30.3) | 72.4 (61.3-83.4) | 33.1 (11.9-54.3) |
| Retired, jobless, or disability insurance (N=479) | 37.1 (31.2-42.9) | 24.9 (20.3-29.5) | 54.8 (42.9-66.7) | 69.6 (56.1-83.1) |
| *Monthly household income* |  |  |  |  |
| <3000CHF (N=110) | 39.4 (29.8-49.1) | 33.9 (32.0-35.8) | 41.9 (33.7-50.2) | 65.5 (57.2-73.8) |
| 3000-4999CHF (N=304) | 38.8 (33.1-44.4) | 17.3 (9.6-24.9) | 56.3 (46.6-65.9) | 44.0 (34.9-53.2) |
| 5000-6999CHF (N=376) | 33.3 (29.0-37.7) | 22.7 (13.9-31.5) | 53.1 (42.3-63.9) | 59.0 (47.9-70.1) |
| 7000-9499CHF (N=457) | 36.1 (31.8-40.4) | 24.6 (17.2-32.0) | 66.1 (60.1-72.2) | 77.3 (66.3-88.2) |
| 9500-13000CHF (N=397) | 37.1 (32.6-41.5) | 19.5 (12.6-26.4) | 61.0 (54.6-67.5) | 44.4 (36.1-52.6) |
| >13000CHF (N=380) | 28.2 (23.3-33.0) | 14.0 (7.5-20.6) | 56.6 (47.2-66.1) | 28.3 (17.8-38.8) |
| *Unadjusted P value for trends* | **<0.001** | 0.93 | 0.05 | 0.28 |
| *Adjusted* P value for trends* | 0.13 | 0.71 | 0.54 | 0.20 |

*Adjusted for job position, smoking status, gender, swiss citizenship, sedentarity, obesity, hypercholesterolemia, diabetes, age, alcool consumption, and eduction level
